# Global trends and research hotspots of EAT-Lancet diet: a bibliometric analysis

**DOI:** 10.3389/fnut.2023.1328351

**Published:** 2024-01-05

**Authors:** Xiaoxiao Lin, Shuai Wang, Yue Gao

**Affiliations:** ^1^Department of Geriatrics, Affiliated Hangzhou First People’s Hospital, School of Medicine, Westlake University, Hangzhou, Zhejiang, China; ^2^Zhejiang Key Laboratory of Traditional Chinese Medicine for the Prevention and Treatment of Senile Chronic Diseases, Hangzhou, China; ^3^School of Public Health, Sir Run Run Shaw Hospital, Zhejiang University School of Medicine, Hangzhou, Zhejiang, China

**Keywords:** EAT-lancet diet, bibliometric analysis, research hotspots, human health, adaptation

## Abstract

The EAT-Lancet diet is a groundbreaking and comprehensive dietary framework that has garnered significant attention in the fields of nutrition, sustainability, and public health. We aimed to conduct a bibliometric study to investigate current status and hotspots in the field of EAT-Lancet diet based on the Web of Science Core Collection (WOSCC) database, and the documents of EAT-Lancet diet published from Jan 1, 2019 to Sep 1.2023 were extracted. The bibliometric and visualized analysis were performed by VOSviewer 1.6.16 and WOSCC Online Analysis Platform. In total, 155 documents from 62 journals were included, and 735 authors from 389 institutions and 53 countries/regions contributed to the field of EAT-Lancet diet. The most productive countries/regions, institutions, authors, and journals were the USA, Wageningen University & Research, Johan Rockström, and Nutrients, respectively. The first high-cited document was published in Lancet and authored by Willett et al. in 2019. This is also the first study about EAT-Lancet diet. The article firstly proposed the “EAT-Lancet Diet” emphasizing balanced, plant-based eating to improve human health while addressing environmental concerns. In conclusion, in the field of EAT-Lancet diet, the main research hotspots and frontiers are the adaptation of EAT-Lancet diet, the composition of EAT-Lancet diet, and the benefits of EAT-Lancet diet for human health. The number of research on the EAT-Lancet diet is currently limited. There is a pressing need for further studies to broaden our understanding of the EAT-Lancet diet and its potential to enhance human health.

## Introduction

The EAT-Lancet diet, also known as the “planetary health diet,” is a groundbreaking and comprehensive dietary framework that has garnered significant attention in the fields of nutrition, sustainability, and public health. Proposed by the EAT-Lancet Commission, a collaboration of experts from around the world, this diet is designed to address two interconnected global challenges: optimizing human health and promoting environmental sustainability (1–5). The EAT-Lancet diet emphasizes a profound shift in our eating habits to achieve a more sustainable and health-conscious way of nourishing ourselves. It goes beyond the traditional understanding of dietary guidelines by considering not only the nutritional needs of individuals but also the environmental impact of our food choices (6–8). This revolutionary dietary approach calls for a significant increase in the consumption of plant-based foods, such as whole grains, fruits, vegetables, legumes, and nuts. It encourages a reduction in the intake of animal-based products, particularly red meat, while allowing moderate amounts of fish and poultry. Added sugars, refined grains, and unhealthy fats are minimized in favor of healthier fats from sources like avocados and nuts. What truly sets the EAT-Lancet diet apart is its dual focus on human well-being and the planet’s health. It recognizes the urgent need to transform our global food systems to align with the United Nations’ Sustainable Development Goals (SDGs) and the Paris Agreement. By adopting this dietary approach, it is believed that we can not only improve individual health but also mitigate the environmental challenges posed by agriculture, including climate change, biodiversity loss, and resource depletion. Moreover, the EAT-Lancet diet is designed to be flexible, adaptable to diverse cultural, economic, and environmental contexts worldwide. It offers a blueprint for healthier eating patterns that can be tailored to local traditions and preferences. The EAT-Lancet diet represents a visionary response to the complex and interconnected issues of nutrition and sustainability. It offers a path toward healthier lives for individuals and a more sustainable future for our planet, making it a topic of great importance and interest in the realms of nutrition, public health, and environmental science (4, 9–13).

The EAT-Lancet diet has garnered significant interest. However, despite the importance of this topic, a comprehensive bibliometric analysis is lacking, one that encapsulates existing publication patterns and forecasts emerging research focal points in this domain. Bibliometric analysis is a systematic and quantitative method used to assess and analyze the scholarly literature related to a specific research topic (14–18). It involves the collection and evaluation of bibliographic data, such as publications, citations, authors, journals, and institutions, to gain insights into the research trends, knowledge networks, and impact of this dietary framework. By employing bibliometric techniques, researchers can uncover key patterns and dynamics in the academic discourse surrounding the EAT-Lancet diet, identify influential authors and institutions, track the evolution of research themes over time, and discern emerging areas of interest and research gaps. This approach may be helpful to both academics and policymakers seeking a comprehensive understanding of the scientific landscape and the dissemination of knowledge in the realm of sustainable and healthy diets. Consequently, our study embarked on a bibliometric evaluation with the objective of identifying the leading edges and hotspots within the realm of the EAT-Lancet diet.

## Methods

### Data sources and search strategies

The primary literature source for this bibliometric analysis was the Web of Science Core Collection (WoSCC), renowned for its comprehensive and authoritative collection of research publications. The specific search terms utilized were (EAT-Lancet OR “planetary health diet” OR “planetary diet”). As the first document about the EAT-Lancet diet was published in 2019 (2), the search results were confined by the publication date from Jan 1, 2019 to Sep 1.2023; with all types of publications related to EAT-Lancet diet, and with no language restriction.

### Bibliometric analysis and visualization

Once the relevant literature was identified and selected, several metrics were analyzed using the WoSCC Online Analysis Platform. This included the annual number of publications, a ranking of the top 10 most productive countries/regions, institutions, authors, and journals, and an identification of the top 20 highly-cited articles related to the “EAT-Lancet” topic. For further in-depth analysis, the data, encompassing details about publication years, authors, countries, regions, institutions, journals, keywords, and references, was downloaded in TXT format with the “Full Record and Cited References” option. Subsequently, VOSviewer software (version 1.6.16) was utilized to visualize various data sets, including co-authorship networks (spanning institutions, countries/regions, and individual authors), citation patterns of journals and references, co-citation networks, and keyword co-occurrence networks. Upon completion, pertinent visualizations were exported to support the findings of the analysis. In the keyword co-occurrence analysis, we merged the synonyms of “sustainable diet” to “sustainable diets” and “impact” to “impacts.”

## Results

### Trends in global publications

**Figure 1:**
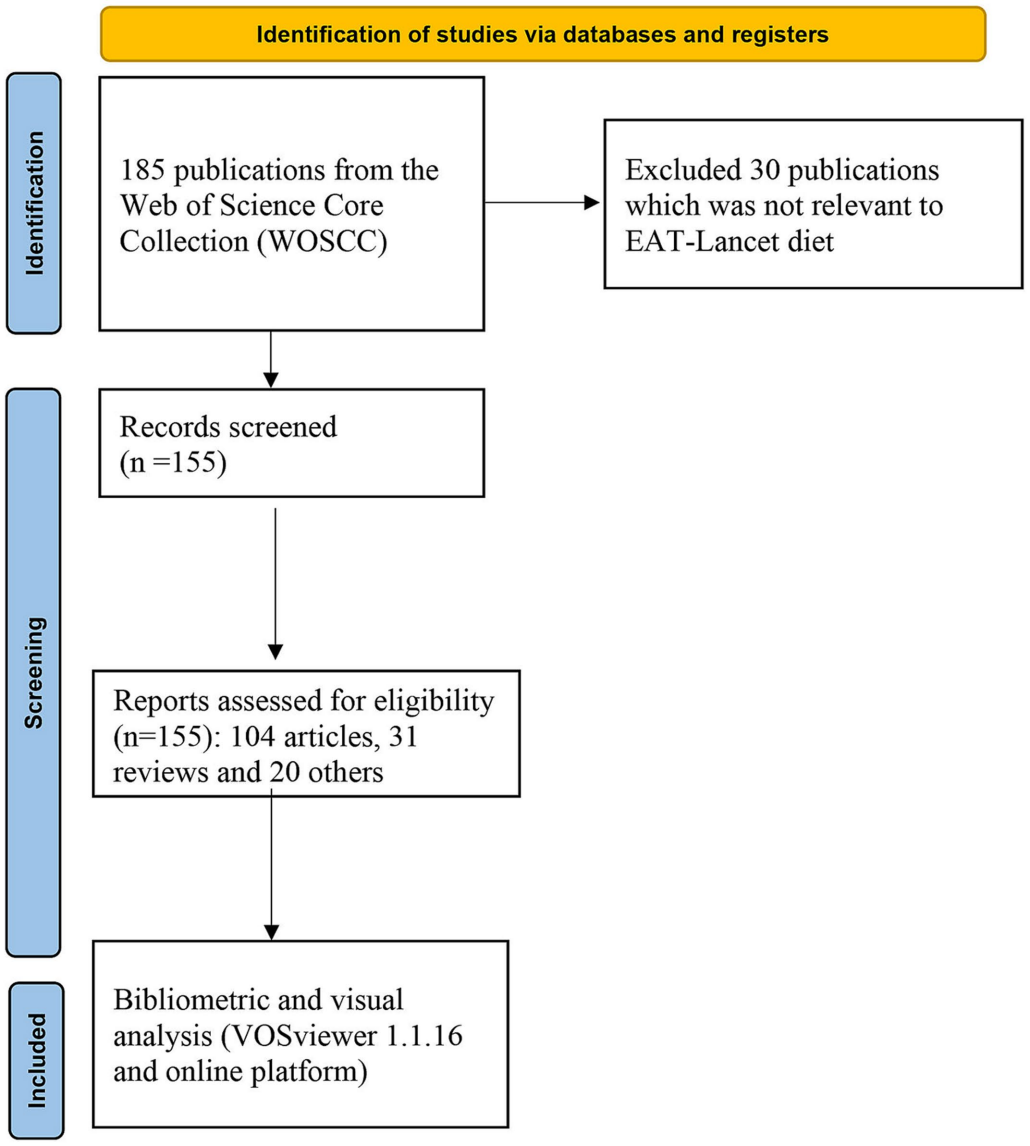
Flow search chart.

**Figure 2:**
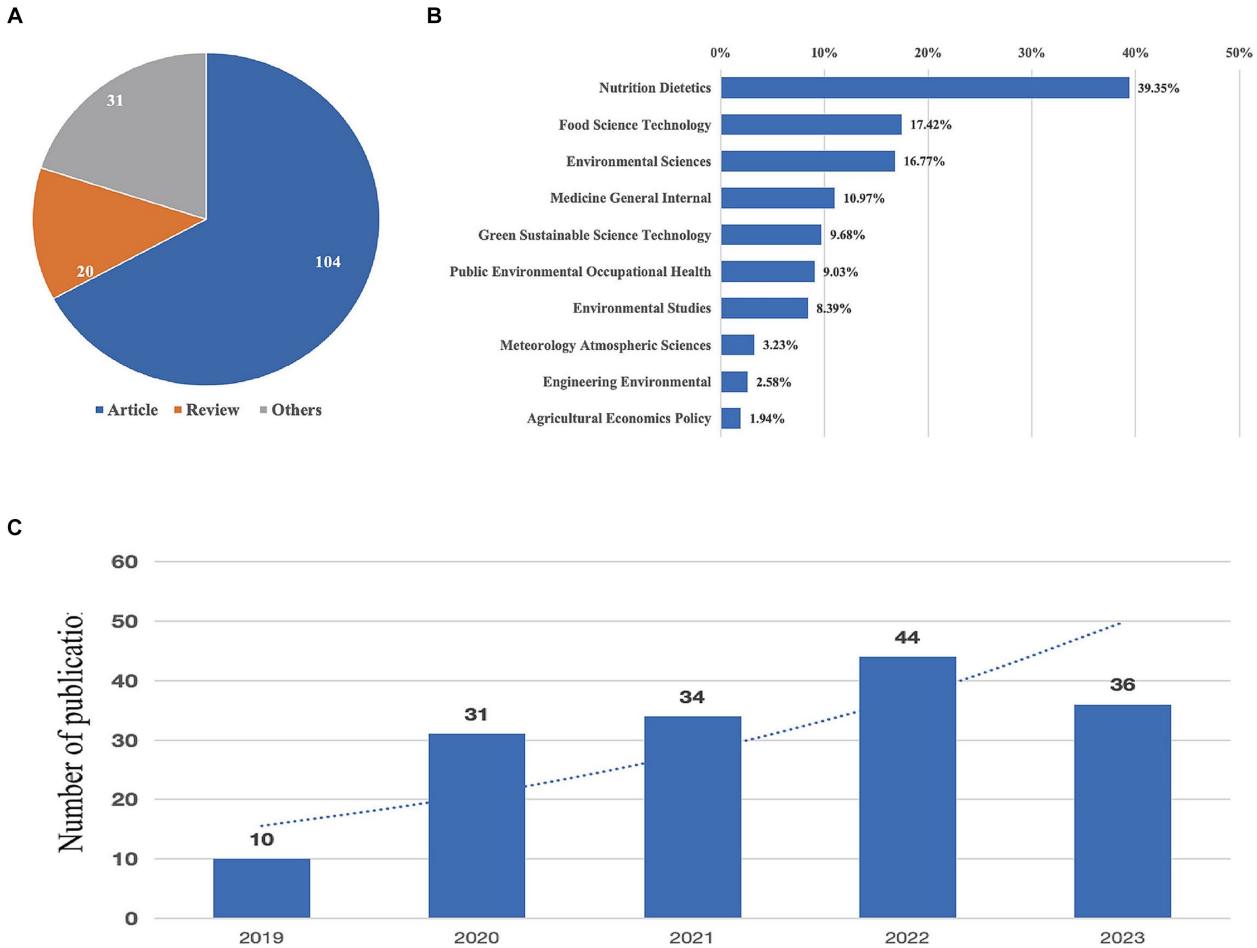
Yearly quantity and literature type of publications.

Based on the provided visual data, a total of 155 documents concerning the topic “EAT-Lancet” were identified in the bibliometric study after screening, which is shown in Figure 1. The breakdown of the types of documents as visualized in Figure 2A, suggests that there were 104 articles (67.1%), 31 reviews (20%), and 20 other types of publications (12.9%). For the subject areas where the documents were primarily published, Figure 2B provides a detailed breakdown. The leading subject category was “Nutrition Dietetics” with 61 documents, accounting for 39.35% of the total publications. This was followed by “Food Science Technology” with 27 documents, representing 17.42%, and “Environmental Sciences” with 26 documents, or 16.67%. Regarding the temporal distribution of publications, Figure 2C depicts the trend from 2019 to 2023. The growth in the number of publications on the “EAT-Lancet” topic is evident, starting with 10 in 2019, more than doubling to 31 in 2020, followed by a consistent rise to 34 in 2021, 44 in 2022, and reaching 36 in 2023. The dotted line in the figure indicates an overall positive trend in the number of publications over the years, emphasizing the increasing importance and recognition of the “EAT-Lancet” topic in the academic and scientific community.

### Analysis of countries or regions, institutions, and authors, journals

**Figure 3:**
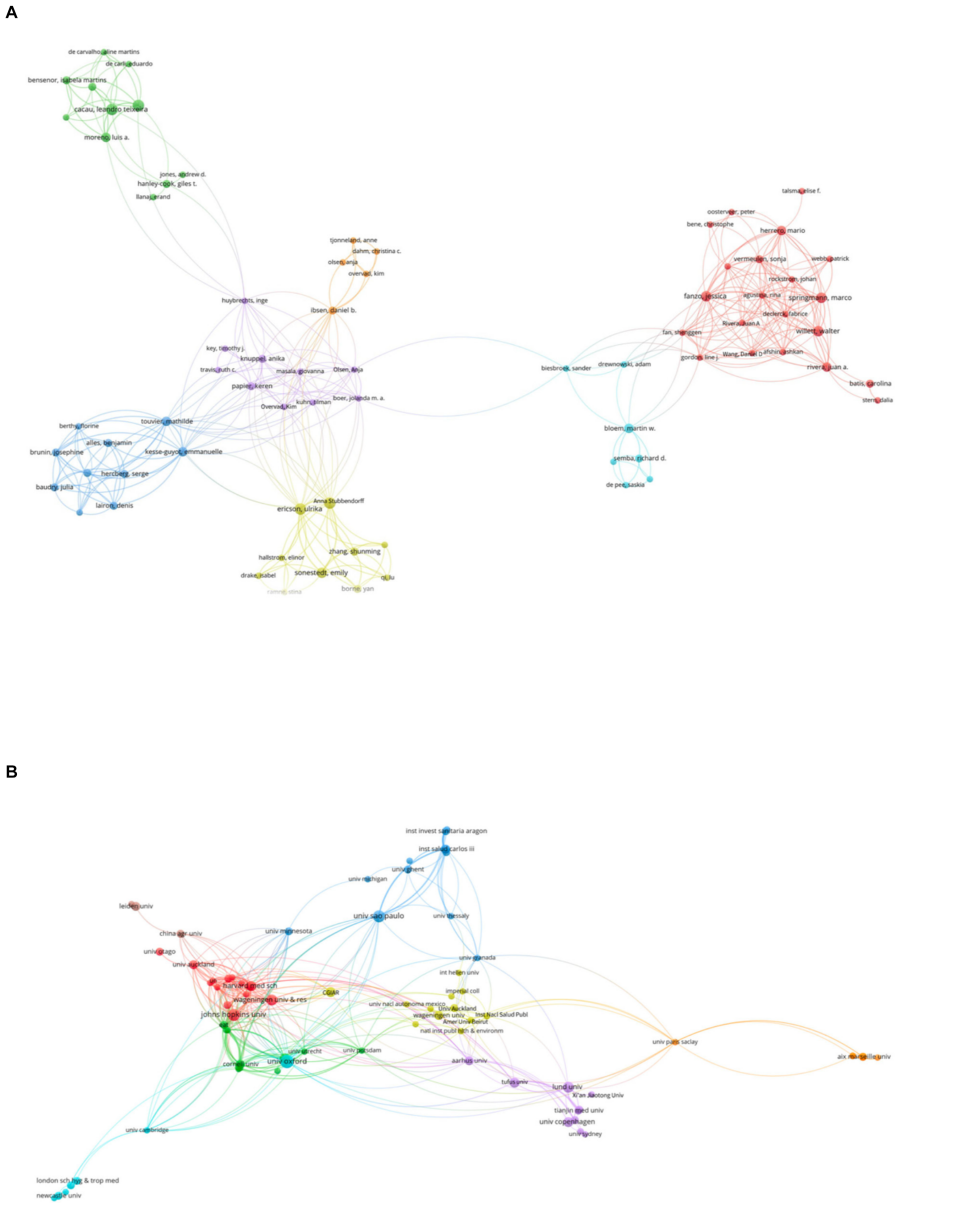
Visualization knowledge maps of authors and institutions.

**Figure 4:**
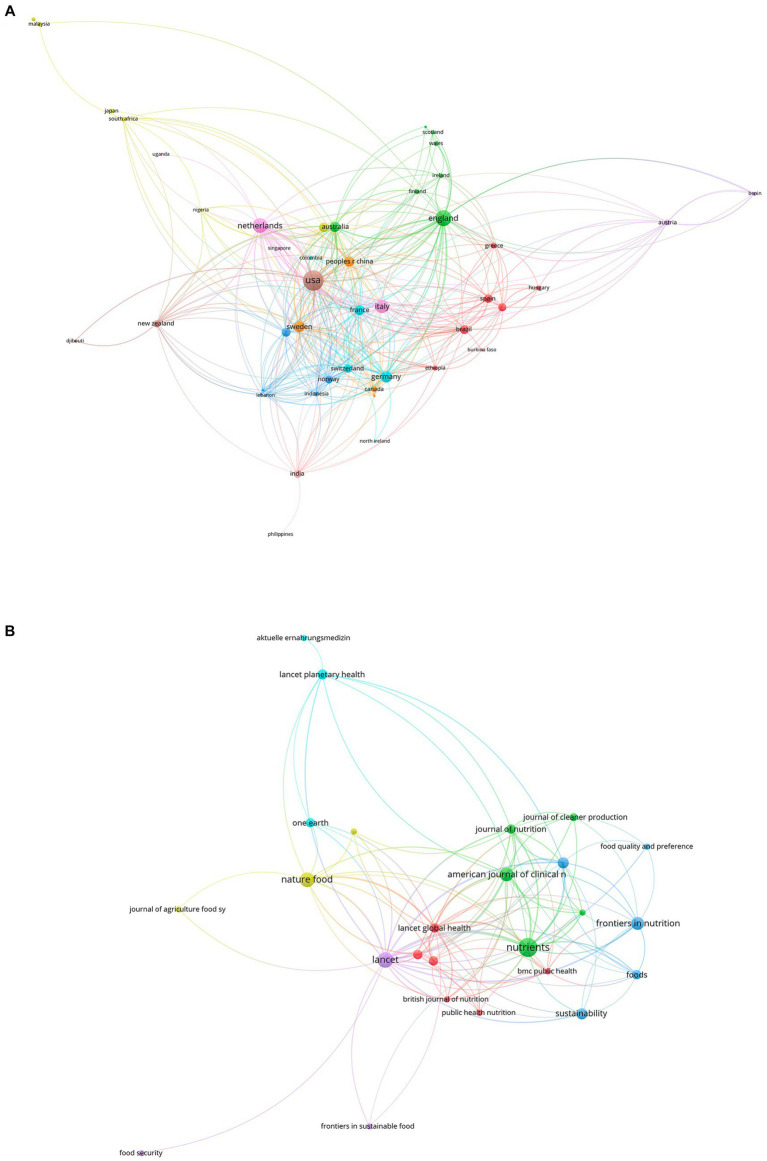
The visualization knowledge map of countries/regions and journals.

In total, 735 authors from 389 institutions and 53 countries/regions contributed to the field of EAT-Lancet diet in 62 journals. On the country front, the USA leads the contribution with 45 publications, gathering a considerable 5,146 citations and an H-index of 17, reflecting both volume and the impact of the research. Following the USA, England with 28 publications and the Netherlands with 23 have been active contributors. While the USA and England are expected leaders given their robust research ecosystems, the significant contribution from the Netherlands suggests a keen interest in the topic regionally. Wageningen University & Research stands out as the top institution in this field, with 11 publications, followed closely by globally renowned institutions like Harvard University with 10 publications and the University of Oxford with 9. When it comes to individual contributors, Johan Rockström tops the list with 6 publications and an impressive 4,046 citations. Ulrika Ericson and Leandro Teixeira Cacau also appear prominent with each having 6 publications. Notably, while Johan Rockström has the same number of publications as Ulrika and Leandro, his citation count is considerably higher. The top three journals that published the most on the EAT-Lancet topic are “Nutrients” from Switzerland with 17 publications, “Lancet” from England boasting a significant 4,130 publications, and “Nature Food” from England with 10 publications. In summary, the EAT-Lancet topic has garnered significant attention from prestigious journals, leading countries in research, globally renowned institutions, and acclaimed researchers. The data underscores the topic’s relevance and the collaborative nature of the research spanning across multiple countries and institutions. The most productive authors, institutions, and countries/regions in the field of EAT-Lancet diet are summarized in Table 1. The network visualization maps of citations of journals, countries/regions, institutions, and authors, are shown in Figures 3, 4 (see Table 2).

**Table 1:** The top productive authors, institutions and countries based on publications.

Items	Publications			
	Ranking	Country	Number	Citations
**Country**	1	USA	45	5,146
	2	England	28	4,552
	3	Netherlands	23	4,369
	4	Italy	20	4,281
	5	Germany	15	4,167
	6	Sweden	14	4,178
	7	Australia	12	4,300
	8	France	12	4,136
	9	Peoples R China	12	84
	10	Denmark	10	122
**Institution**	1	Wageningen University & Research	11	4,213
	2	Harvard university	10	4,273
	3	University of Oxford	9	4,386
	4	CGIAR	8	4,381
	5	Instituto Nacional De Salud Publica	8	4,082
	6	Johns Hopkins University	8	4,126
	7	Universidade De São Paulo	7	57
**Author**	1	Johan Rockström	6	4,046
	2	Ulrika Ericson	6	57
	3	Leandro Teixeira Cacau	6	56
	4	Brent Loken	6	4,046
	5	Dirce Maria Marchioni	6	56
	6	Anna Stubbendorff	6	57
	7	Walter Willett	5	4,072
	8	Juan A Rivera	5	4,079

**Table 2:** The top productive journals.

Ranking	Journal name	Country	Counts	Citation
1	Nutrients	Switzerland	17	198
2	Lancet	England	11	4,130
3	Nature Food	England	10	173
4	American Journal of Clinical Nutrition	USA	9	78
5	Frontiers in Nutrition	Switzerland	8	183
6	European Journal of Nutrition	Germany	6	20
7	Sustainability	Switzerland	6	71
8	Lancet Planetary Health	England	5	54

### Analysis of highly-cited and co-cited publications

**Figure 5:**
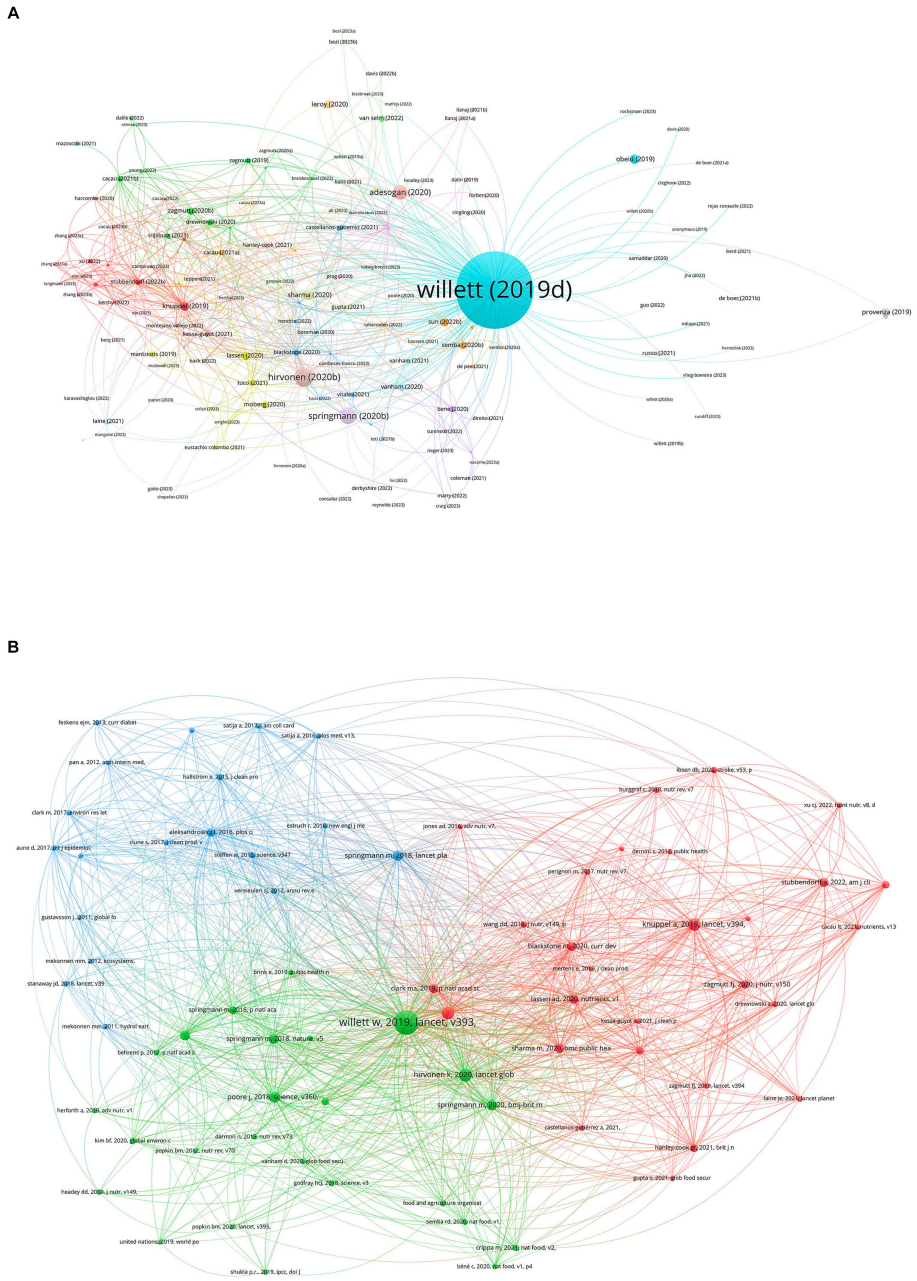
The visualized citations of highly-cited and co-cited publications.

The network visualization maps of citations and co-cited documents are displayed in Figure 5. The attributes of the 20 most-cited publications are encapsulated in Table 3 (1–3, 6, 8, 19–33). The first high-cited document was published in Lancet and authored by Willett et al. (2). This is also the first study about EAT-Lancet diet. The article firstly proposed the “EAT-Lancet Diet” emphasizing balanced, plant-based eating to improve human health while addressing environmental concerns. It stresses the need for transforming food systems, considering global and regional perspectives, reducing environmental impacts, promoting food equity, and implementing policy changes for a more sustainable and nutritious future. The second high-cited publication was published in Lancet Global Health by Hirvonen et al. (21). They conducted a global analysis of the affordability of the EAT-Lancet reference diet. The EAT-Lancet reference diet is a dietary framework designed to promote both human health and environmental sustainability. It emphasizes a balanced diet with a focus on plant-based foods while limiting the consumption of red meat and sugar. The research aimed to assess whether the EAT-Lancet reference diet is affordable for people in different regions around the world. Affordability is a crucial factor in determining whether individuals and households can access and adopt a particular dietary pattern. Key findings and implications from the study may include insights into the affordability of the EAT-Lancet reference diet across various countries and income levels. It can inform discussions and policy considerations related to promoting sustainable and healthy diets globally. The third high-cited publication was published in BMJ and authored by Springmann et al. (28). They performed a modeling analysis that focuses on evaluating the healthiness and sustainability of national and global food-based dietary guidelines (FBDGs). FBDGs are recommendations provided by governments and health organizations regarding what individuals and populations should eat for optimal health. The research uses mathematical models to assess the nutritional quality of dietary guidelines from various countries and regions, including the EAT-Lancet diet, considering factors such as their impact on human health and their environmental sustainability. The study aims to provide insights into whether these guidelines are aligned with both health and sustainability goals. Key findings and implications from the study may include assessments of the strengths and weaknesses of different dietary guidelines and recommendations for improving the alignment of FBDGs with health and sustainability objectives. The fourth high-cited publication was published in Global Food Security and authored by Adesogan et al. (19). They explore the role of animal source foods in the context of sustainability and nutrition. It emphasizes that perspectives on this issue vary and depend on one’s viewpoint. In the context of the EAT-Lancet diet, which encourages a shift towards plant-based eating for sustainability reasons, this article may provide an alternative perspective. It suggests that animal source foods can play a role in addressing malnutrition and may be part of a sustainable food system when managed responsibly. However, the article acknowledges that different viewpoints exist and highlights the importance of considering various perspectives when discussing the role of animal source foods in diets and sustainability. The fifth high-cited publication was published in Frontiers in Nutrition and authored by Obeid et al. (25). They explore the important role of vitamin B12 intake primarily from animal-based foods. In the context of the EAT-Lancet diet, which emphasizes a reduction in animal product consumption for sustainability reasons, this article provides insights into the potential health implications of lower vitamin B12 intake. Vitamin B12 is primarily found in animal products, and reduced consumption of these foods can lead to vitamin B12 deficiency, which has health implications. The article likely discusses the importance of monitoring vitamin B12 levels and the potential need for supplementation or alternative dietary sources in plant-based diets to ensure adequate intake and avoid health issues associated with deficiency. It highlights the complexity of dietary choices and their impact on nutrient intake and health, especially when considering diets that limit animal-based foods like those promoted by the EAT-Lancet diet. For the co-cited documents, the top 2 highest co-cited studies were consistent with the highest cited publications. The third highest co-cited document was published in Lancet by Afthin et al. in 2019 (34). In this systematic analysis, they investigated the influence of poor diets on non-communicable diseases (NCDs) across 195 countries. Utilizing a comparative risk assessment method, it was found that in 2017, dietary risk factors contributed to 11 million deaths and 255 million disability-adjusted life-years (DALYs). The leading dietary culprits included high sodium intake, insufficient whole grains, and low fruit consumption. Despite some data uncertainties due to varied sources, the study emphasizes the critical role of diet in global health and underscores the need for evidence-based dietary interventions. The fourth highest co-cited document was published in Lancet by knuppel et al. (1). It is the first study about EAT-Lancet diet and main health outcomes. In this study conducted on 46,069 participants from the UK involved in the EPIC-Oxford research, the EAT-Lancet Commission’s 2019 universal reference diet, aimed at promoting human and environmental health, was evaluated. Using food frequency questionnaires from 1993 to 2001, participants were scored between 0–14 based on their adherence to 14 key dietary recommendations. Results indicated that high adherence to the EAT-Lancet score corresponded to a 28% reduced risk of ischemic heart disease and a 59% reduced risk of diabetes. However, there was no clear link between the score and the risk of stroke or overall mortality. The study suggests that the diet’s benefits are cumulative from multiple recommendations, with the majority of participants adhering to recommendations for poultry, eggs, fish, legumes, and fats. The high adherence might also reflect the cohort’s large vegetarian population and could be a marker for an overall healthy lifestyle. The fifth highest co-cited document was published in science by Springmann in 2020 (35). Their research reveals significant variation, up to 50-fold, in environmental impact among producers of the same product, suggesting opportunities for environmental mitigation. However, achieving these reductions is complex due to trade-offs, diverse methods available to producers, and supply chain interactions. Interestingly, even the lowest-impact animal products tend to have greater environmental impacts than vegetable substitutes, emphasizing the potential environmental benefits of dietary shifts. The study advocates for producers to actively monitor and communicate their environmental impacts, and to flexibly choose from various practices to meet environmental goals.

**Table 3:** The top 20 most highly cited references.

Rank	Title	Journal	Citations	Year	First author
1	Food in the Anthropocene: the EAT-Lancet Commission on healthy diets from sustainable food systems	Lancet	4,040	2019	Walter Willett
2	Affordability of the EAT-Lancet reference diet: a global analysis	Lancet Global Health	225	2020	Kalle Hirvonen
3	The healthiness and sustainability of national and global food based dietary guidelines: modelling study	BMJ	195	2020	Marco Springmann
4	Animal source foods: Sustainability problem or malnutrition and sustainability solution? Perspective matters	Global Food Security	146	2020	Adegbola Tolulope Adesogan
5	Vitamin B12 Intake from Animal Foods, Biomarkers, and Health Aspects	Frontiers In Nutrition	68	2019	Rima Obeid
6	A comparison of the Indian diet with the EAT-Lancet reference diet	Bmc Public Health	67	2020	Manika Sharma
7	EAT-Lancet score and major health outcomes: the EPIC-Oxford study	Lancet	56	2019	Anika Knuppel
8	Should dietary guidelines recommend low red meat intake?	Critical Reviews in Food Science And Nutrition	54	2020	Frédéric Leroy
9	Dietary change in high-income nations alone can lead to substantial double climate dividend	Nature Food	48	2022	Zhongxiao Sun
10	Development of a Danish Adapted Healthy Plant-Based Diet Based on the EAT-Lancet Reference Diet	Nutrients	47	2020	Anne D Lassen
11	Bambara Groundnut: An Underutilized Leguminous Crop for Global Food Security and Nutrition	Frontiers In Nutrition	41	2020	Xin Lin Tan
12	Benchmarking the Swedish Diet Relative to Global and National Environmental Targets-Identification of Indicator Limitations and Data Gaps	Sustainability	35	2020	Emma Moberg
13	Circularity in animal production requires a change in the EAT-Lancet diet in Europe	Nature Food	34	2022	Benjamin van Selm
14	Adoption of the ‘planetary health diet’ has different impacts on countries’ greenhouse gas emissions	Nature Food	34	2020	Richard D Semba
15	The EAT-Lancet Commission’s Dietary Composition May Not Prevent Noncommunicable Disease Mortality	Journal of Nutrition	34	2020	Francisco J Zagmutt
16	Treenuts and groundnuts in the EAT -Lancet reference diet: Concerns regarding sustainable water use	Global Food Security	34	2020	Davy Vanham
17	Five priorities to operationalize the EAT-Lancet Commission report	Nature Food	31	2020	Christophe Béné
18	Co-benefits from sustainable dietary shifts for population and environmental health: an assessment from a large European cohort study	Lancet Planetary Health	30	2021	Laine JE
19	Comparing the Recommended Eating Patterns of the EAT-Lancet Commission and Dietary Guidelines for Americans: Implications for Sustainable Nutrition	Current Developments In Nutrition	28	2020	Nicole Tichenor Blackstone
20	Analyzing the affordability of the EAT-Lancet diet	Lancet Global Health	28	2020	Adam Drewnowski

### Analysis of keywords

**Figure 6:**
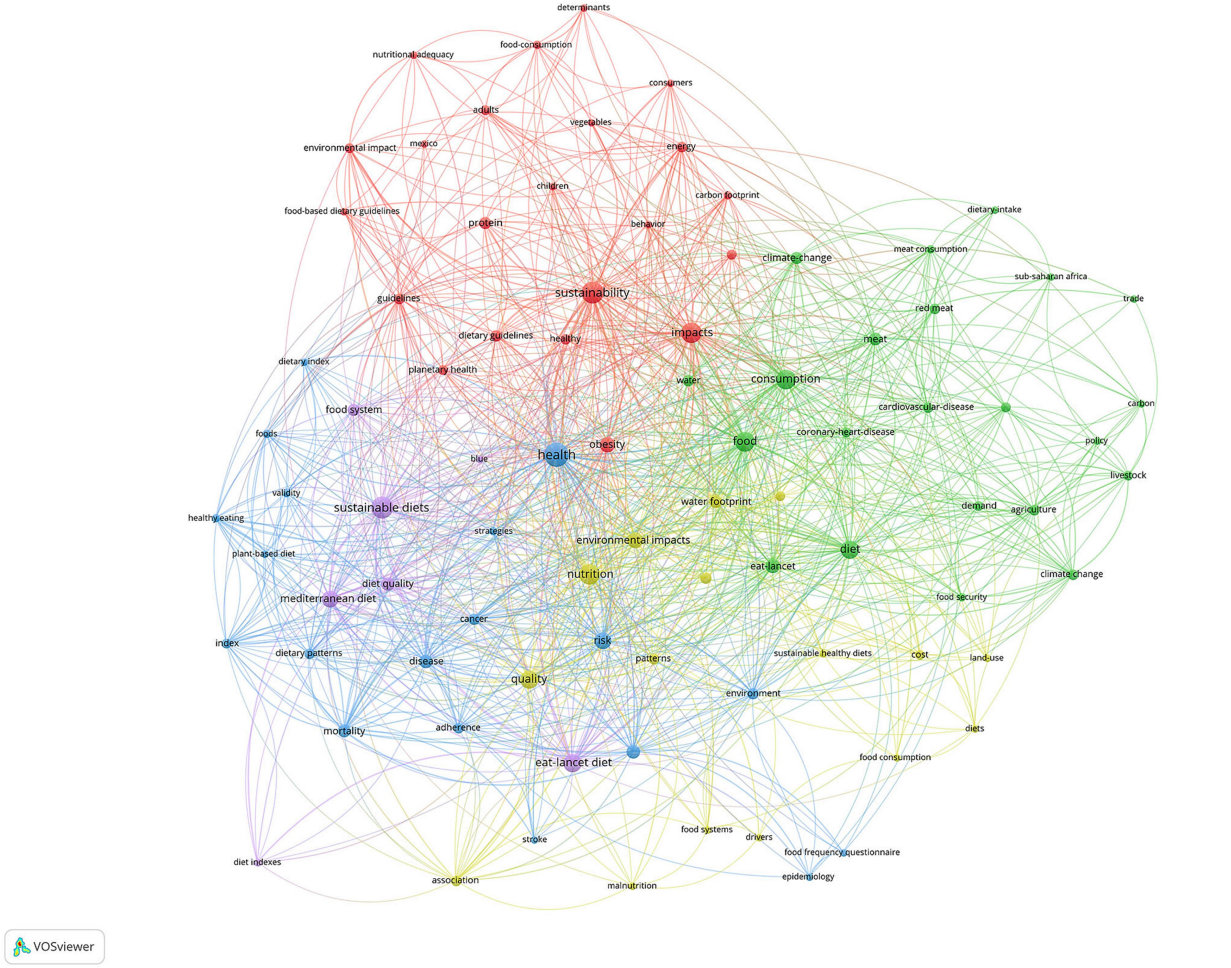
Visualization of keyword co-occurrence analysis.

Figure 6 displayed the network visualization maps of co-occurrence of keywords, which could be classified in four clusters, including: Health impacts and ASPECTS that diets exert on human well-being. This domain covers the gamut from generic health and diet quality to specific ailments like coronary heart diseases and malnutrition. Dietary practices and behaviors, which delve into consumption patterns, food frequency, and tools to measure these behaviors such as the food frequency questionnaire. Dietary patterns and types that include prominent diets like the Mediterranean and Eat-Lancet diets, along with broader themes like dietary guidelines, patterns, and the evolving concept of sustainable diets. Intertwined with these dietary considerations are pressing Environmental Aspects. Key points here encapsulate the environmental impact of dietary choices, highlighting metrics like the carbon and water footprints and the overarching shadow of climate change. Sustainability and global issues emerge as critical, shedding light on the symbiosis between planetary health, food security, and nutritional adequacy.

## Discussion

### General information

To the best of our knowledge, this is the first comprehensive bibliometric analysis of the EAT-Lancet diet. The EAT-Lancet diet represents a transformative step in the field of nutrition, sustainability, and global food systems. It emerged as a critical topic of research and discourse, seeking to address both human health and planetary health, aiming to devise a dietary pattern that would not only optimize health outcomes but also reduce the environmental footprint of our food choices. The data analysis sheds light on the considerable attention the EAT-Lancet topic has garnered from the research community. In our bibliometric analysis, a total of 155 publications addressing the EAT-Lancet diet span across prestigious journals such as “Nutrients,” “Lancet,” and “Nature Food.” The USA leads the global contributions with 45 publications, reflecting the nation’s pivotal role in advancing research on this topic. Notably, 53 countries have made significant contributions, indicating a broad international interest. From an institutional perspective, Wageningen University & Research has been at the forefront, closely followed by globally acclaimed institutions like Harvard University and the University of Oxford. These institutions have not just contributed in volume but have also made impactful contributions as denoted by their high citation counts. On the individual front, Johan Rockström stands out with 6 publications, amassing an impressive 4,046 citations, indicating the broad reach and influence of his work. A pivotal publication from Lancet, authored by Willett et al. deserves special mention. This work was the first to propose the concept of the “EAT-Lancet Diet.” It brought forth the idea of a balanced, primarily plant-based diet aimed at improving human health while simultaneously addressing environmental sustainability. The article emphasized the urgent need for a shift in global food systems, considering both global and regional perspectives. It called for reduced environmental impacts, the promotion of food equity, and the importance of implementing policy changes to drive a sustainable and nutritious future. In essence, the EAT-Lancet diet topic encapsulates a holistic vision of the future of food - one that intertwines human health, environmental sustainability, and global collaboration. The significant contributions from leading countries, institutions, and researchers underscore the topic’s global relevance and the collaborative nature of the research.

### Hotspots and frontiers

On the basis of publications of EAT-Lancet diet, highly-cited publications, and important keywords with high frequency, the research hotspots in the field of EAT-Lancet diet were summarized as follows:

#### The adaptation of EAT-Lancet diet

Adapting the EAT-Lancet diet to diverse cultural and regional contexts is a hotspot (22, 36–41). Traditional diets are deeply rooted in cultural practices, and a one-size-fits-all approach may not be feasible. Customizing the diet to align with cultural preferences and food availability while maintaining sustainability goals is a challenge. Many studies aimed to develop different types of diet based on EAT-Lancet diet to adapt to the needs of different cultures and regions. For example, in a study comparing the environmental impacts of two dietary patterns (42), the Italian-Mediterranean (EAT-IT) based on the “Planetary diet” and the Italian Dietary Guidelines (IDG), it was found that the EAT-IT diet had a significantly lower carbon footprint (*CF*) than the IDG. However, there was no notable difference in their water footprints (WF). Protein-rich foods were major contributors to both *CF* and WF in both diets. Environmental outcomes were further impacted by choices like opting for frozen over fresh foods, imported fruits over local ones, greenhouse vegetables over seasonal varieties, and processed legume foods over unprocessed legumes. The EAT-IT diet is more carbon sustainable, but individual food choices significantly affect its environmental footprint. Lassen et al. (22) aimed to design a healthy plant-based diet tailored for Denmark using the global EAT-Lancet reference diet. Initially, the EAT-Lancet diet was adjusted based on available Danish foods. It was then further refined to match national dietary guidelines and current consumption trends, incorporating processed foods, discretionary items, and beverages. This adapted diet met most nutrient recommendations for individuals aged 6–65 years, excluding vitamin D and iodine. The research highlighted the importance of emphasizing legumes, nuts, seeds, fruits, dark green vegetables, whole grains, and vegetable oils, while reducing meat consumption, offering guidance for future sustainable dietary guidelines. Nomura et al. assessed the Japanese diet in relation to the global EAT-Lancet Commission’s Planetary Health Diet (PHD), particularly focusing on protein intake across various age groups. Using data from the Japan National Health and Nutrition Survey 2019 (43), it was found that while most food group intakes exceeded the PHD’s global reference across all age groups, only red meat intake significantly surpassed its upper limit, particularly in those in their 40s. Despite this, protein consumption in Japan remains within recommended limits. As seen in various Western regions, the Japanese diet has an excessive red meat intake compared to the PHD’s reference. However, the overall protein intake is within the national guidelines. The study underscores the potential of the PHD as an eco-friendly and healthy option for Japan’s aging population. It highlights the need for sustainable dietary guidelines, better nutrition education, and an environment that promotes healthier food choices.

#### The composition of EAT-Lancet diet

A key advantage of the EAT-Lancet diet is its emphasis on sustainability (44–50). By promoting reduced meat consumption and a shift towards plant-based foods, it aims to reduce greenhouse gas emissions, land use, and water use associated with food production. The diet is rich in vegetables, fruits, whole grains, legumes, and nuts. Such a nutrient-rich diet can help reduce the risk of chronic diseases like heart disease, diabetes, and certain cancers. It recommends diverse food sources which can help address global issues of not just overnutrition (like obesity) but also undernutrition. However, some researchers believed that that the diet might not provide adequate amounts of certain nutrients sourced mainly from animal products, such as vitamin B12, iron, calcium, and omega-3 fatty acids. Though these concerns can be addressed with fortified foods and supplements, it remains a point of contention.

#### The benefits of EAT-Lancet diet for human health

The EAT-Lancet diet has emerged as a global benchmark for optimal health, addressing critical health conditions while also championing environmental sustainability through lower carbon emissions and prudent resource use. Its impact has been the subject of various research efforts, particularly concerning diseases like diabetes, cardiovascular diseases (CVDs), and cancer (51–58). For instance, an insightful study delved into how closely following the EAT-Lancet diet influenced the occurrence and death rates from lung cancer (54). This research, based on data from the Prostate, Lung, Colorectal, and Ovarian (PLCO) trial involving 101,755 American adults, sought to clarify previously ambiguous connections between the diet and lung cancer. The findings were compelling, revealing that strict adherence to the EAT-Lancet diet corresponded to a substantial reduction in both the development and fatality rates of lung cancer, especially prevalent in cases of non-small-cell lung cancer. This suggests that maintaining the EAT-Lancet diet could potentially lower lung cancer risks. Colizzi and colleagues (59) focused on creating a diet score reflecting the Healthy Reference Diet (HRD) and evaluating its correlation with cardiovascular outcomes and ecological repercussions, drawing on data from 35,496 individuals in the EPIC-NL study. The results indicated that participants who closely followed the HRD registered lower probabilities of experiencing cardiovascular disease (14% reduction), coronary heart disease (12% reduction), and stroke (11% reduction). Another research initiative involving 24,494 participants investigated the link between the largely plant-focused EAT-Lancet diet (58), known for its dual benefits on human health and ecological sustainability, and the likelihood of T2DM. Following a median 24.3-year observation period, the study noted a striking 18% risk reduction for T2DM among those most committed to the EAT-Lancet diet compared to their least compliant counterparts. Interestingly, the diet’s effectiveness seemed unhindered by genetic factors predisposing individuals to T2DM, suggesting its protective role regardless of genetic liabilities. One such research effort focused on understanding the broader health repercussions of the globally advocated EAT-Lancet diet led to the formulation of a novel dietary index to measure compliance. This study, utilizing the Malmö Diet and Cancer cohort’s data, comprising 22,421 Swedes aged 45–73 years, examined dietary habits over roughly two decades (56). The conclusions were significant: high fidelity to the EAT-Lancet diet was associated with a 25% decline in the risk of death from all causes, with remarkable reductions concerning cancer and cardiovascular-related deaths. These findings highlight the extensive health advantages of the EAT-Lancet diet, bolstering the case for its integration into sustainable eating protocols worldwide. Moreover, certain studies have indicated that higher adherence to the EAT-Lancet diet correlates with decreased overall mortality risks (1, 60).

However, the EAT-Lancet diet has sparked significant debate and criticism. Critics argue that its one-size-fits-all approach overlooks cultural and individual dietary needs, potentially leading to nutritional deficiencies in populations accustomed to different eating habits (61, 62). Beal et al. (5) evaluated the estimated micronutrient shortfalls of the EAT–Lancet diet, and found that the EAT–Lancet diet might not provide adequate nutrients, especially in micronutrients like iron, calcium, and zinc, potentially leading to some public health issues. This diet, focusing on minimally processed plant foods and low in animal sources, may require adjustments, such as increasing nutrient-dense foods like fish, shellfish, seeds, eggs, and beef, and decreasing foods high in phytate. Achieving dietary nutrient adequacy sustainably for the global population involves complex trade-offs between environmental preservation, reducing non-communicable diseases (NCDs), and nutrient adequacy. This calls for further analysis using dietary optimization modelling and life cycle assessments. The study suggests moving away from a one-size-fits-all planetary health diet towards context-specific guidelines, considering local data, cultural contexts, and environmental conditions. It emphasizes the need for inclusive approaches involving all stakeholders and underscores the integral link between human health and environmental preservation in addressing these global challenges. In addition, concerns have also been raised about the feasibility of its widespread adoption, given the varying agricultural capabilities and economic conditions across regions. Hirvonen et al. (21) investigated the financial feasibility of the EAT–Lancet diet. Analyzing food price and income data from 159 countries, the study found that the most affordable EAT–Lancet diets averaged $2.84 per day in 2011, with fruits and vegetables being the most expensive component. While affordable in high-income countries, this diet is beyond the financial reach of the world’s poor, with an estimated 1.58 billion people unable to afford it. The cost of the EAT–Lancet diet also exceeds that of a minimum-cost diet meeting essential nutrient requirements by about 60% on average. This indicates that adopting the EAT–Lancet diet widely would require a combination of higher incomes, nutritional assistance, and lower food prices, alongside both local and systemic interventions to reduce the cost of healthier foods. Additionally, there’s skepticism about the diet’s impact on global food systems, with some suggesting it could exacerbate food insecurity in less affluent areas. Proponents, however, emphasize its potential benefits for health and the environment, advocating it as a necessary shift in the face of climate change and a growing global population. Despite these controversies, the EAT-Lancet diet continues to influence discussions on sustainable diets and global health.

There were some limitations in our study. We utilized the WoSCC database, and other databases like Embase and Pubmed are not used, due to the VOSviewer software’s inability to process, analyze, and visually represent co-citation maps from their data. The total volume of publications concerning the EAT-Lancet diet is comparatively limited, indicating an immediate need for additional research. Expanding our understanding of the EAT-Lancet diet’s impact on human health is critical, as is leveraging this dietary approach for health enhancement purposes.

In conclusion, our study is the first bibliometric analysis of EAT-Lancet diet. The main research hotspots and frontiers are the adaptation of EAT-Lancet diet, the composition of EAT-Lancet diet, and the benefits of EAT-Lancet diet for human health. The number of research on the EAT-Lancet diet is currently limited. There is a pressing need for further studies to broaden our understanding of the EAT-Lancet diet and its potential to enhance human health.

## Data availability statement

The original contributions presented in the study are included in the article/supplementary material, further inquiries can be directed to the corresponding authors.

## Author contributions

XL: Data curation, Formal analysis, Methodology, Project administration, Supervision, Validation, Writing – original draft, Writing – review & editing. SW: Data curation, Formal analysis, Methodology, Project administration, Supervision, Validation, Writing – original draft, Writing – review & editing. YG: Formal analysis, Funding acquisition, Project administration, Resources, Validation, Visualization, Writing – original draft, Writing – review & editing.
