# Corrigendum: Global trends and research hotspots of EAT-Lancet diet: a bibliometric analysis

**DOI:** 10.3389/fnut.2024.1454219

**Published:** 2024-08-13

**Authors:** Xiaoxiao Lin, Shuai Wang, Yue Gao

**Affiliations:** ^1^Department of Geriatrics, Affiliated Hangzhou First People's Hospital, School of Medicine, Westlake University, Hangzhou, Zhejiang, China; ^2^Zhejiang Key Laboratory of Traditional Chinese Medicine for the Prevention and Treatment of Senile Chronic Diseases, Hangzhou, China; ^3^School of Public Health, Sir Run Run Shaw Hospital, Zhejiang University School of Medicine, Hangzhou, Zhejiang, China

**Keywords:** EAT-lancet diet, bibliometric analysis, research hotspots, human health, adaptation

In the published article, there was an error in Figure 3. There were missing names on the nodes. The corrected Figure 3 and its caption appear below.

**Figure 3:**
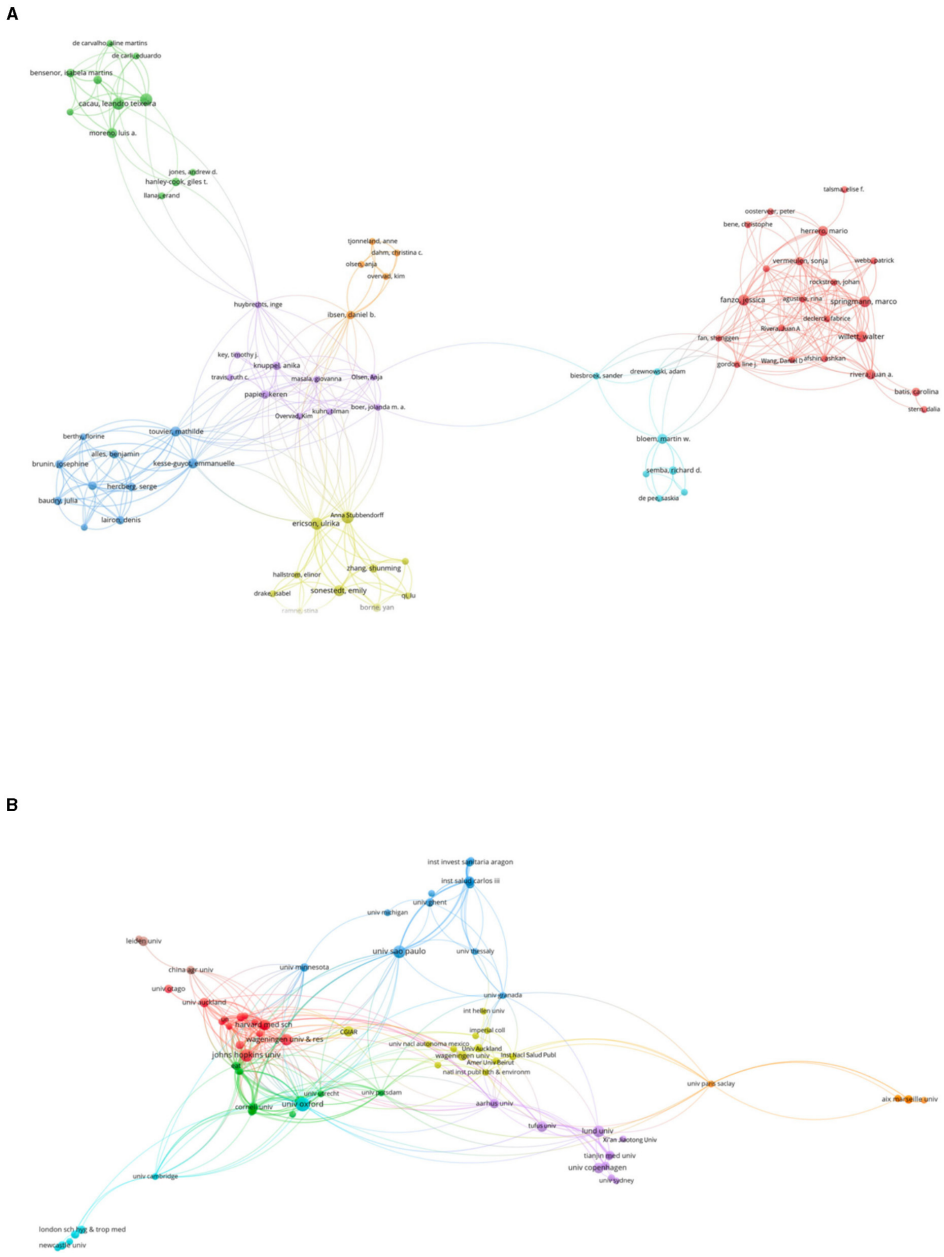
Visualization knowledge maps of authors and institutions.

The authors apologize for this error and state that this does not change the scientific conclusions of the article in any way. The original article has been updated.

